# Detection of *BRAF* splicing variants in plasma-derived cell-free nucleic acids and extracellular vesicles of melanoma patients failing targeted therapy therapies

**DOI:** 10.18632/oncotarget.27790

**Published:** 2020-11-03

**Authors:** Michael E. Clark, Helen Rizos, Michelle R. Pereira, Ashleigh C. McEvoy, Gabriela Marsavela, Leslie Calapre, Katie Meehan, Olivia Ruhen, Muhammad A. Khattak, Tarek M. Meniawy, Georgina V. Long, Matteo S. Carlino, Alexander M. Menzies, Michael Millward, Melanie Ziman, Elin S. Gray

**Affiliations:** ^1^School of Medical and Health Sciences, Edith Cowan University, Joondalup, Western Australia, Australia; ^2^School of Biomedical Science, University of Western Australia, Crawley, Western Australia, Australia; ^3^Faculty of Medicine, Health and Human Sciences, Macquarie University, Sydney, New South Wales, Australia; ^4^Westmead Institute for Cancer Research, The University of Sydney, Sydney, New South Wales, Australia; ^5^Melanoma Institute Australia, Sydney, New South Wales, Australia; ^6^Department of Otorhinolaryngology, Head and Neck Surgery, Chinese University of Hong Kong, Hong Kong; ^7^School of Medicine, The University of Western Australia, Crawley, Western Australia, Australia; ^8^Department of Medical Oncology, Fiona Stanley Hospital, Murdoch, Western Australia, Australia; ^9^Department of Medical Oncology, Sir Charles Gairdner Hospital, Nedlands, Western Australia, Australia; ^10^Sydney Medical School, The University of Sydney, Sydney, New South Wales, Australia; ^11^Mater Hospital, North Sydney, New South Wales, Australia

**Keywords:** melanoma, targeted therapy, drug resistance, *BRAF* splicing, extracellular vesicles

## Abstract

The analysis of plasma circulating tumour nucleic acids provides a non-invasive approach to assess disease burden and the genetic evolution of tumours in response to therapy. *BRAF* splicing variants are known to confer melanoma resistance to BRAF inhibitors. We developed a test to screen cell-free RNA (cfRNA) for the presence of *BRAF* splicing variants. Custom droplet digital PCR assays were designed for the detection of *BRAF* splicing variants p61, p55, p48 and p41 and then validated using RNA from cell lines carrying these variants. Evaluation of plasma from patients with reported objective response to BRAF/MEK inhibition followed by disease progression was revealed by increased circulating tumour DNA (ctDNA) in 24 of 38 cases at the time of relapse. Circulating *BRAF* splicing variants were detected in cfRNA from 3 of these 38 patients; two patients carried the BRAF p61 variant and one the p55 variant. In all three cases the presence of the splicing variant was apparent only at the time of progressive disease. *BRAF* p61 was also detectable in plasma of one of four patients with confirmed *BRAF* splicing variants in their progressing tumours. Isolation and analysis of RNA from extracellular vesicles (EV) from resistant cell lines and patient plasma demonstrated that *BRAF* splicing variants are associated with EVs. These findings indicate that in addition to plasma ctDNA, RNA carried by EVs can provide important tumour specific information.

## INTRODUCTION

Mutations in the serine/threonine kinase BRAF are found in 40% of melanomas [1]. The most prevalent mutation in melanoma is *BRAF* V600E, which constitutively activates downstream mitogen-activated protein kinase (MAPK) signalling. Introduction of single agent BRAF mutant inhibitors (BRAFi), such as vemurafenib and dabrafenib, revolutionised the treatment of metastatic melanomas [2, 3]. Furthermore, the combination of BRAF and MEK inhibitors (such as dabrafenib and trametinib, or vemurafenib and cobimetinib) improved tumour response rate and progression-free survival, while attenuating some of the serious adverse events observed with monotherapy [4, 5]. However, even with combination therapies, resistance to MAPK inhibition remains a major challenge in clinical care, with the majority of patients progressing within 10 months [6]. Identification of the mechanism of resistance to targeted therapies in individual patients may offer new insights into strategies for overcoming resistance.

**Figure 1:**
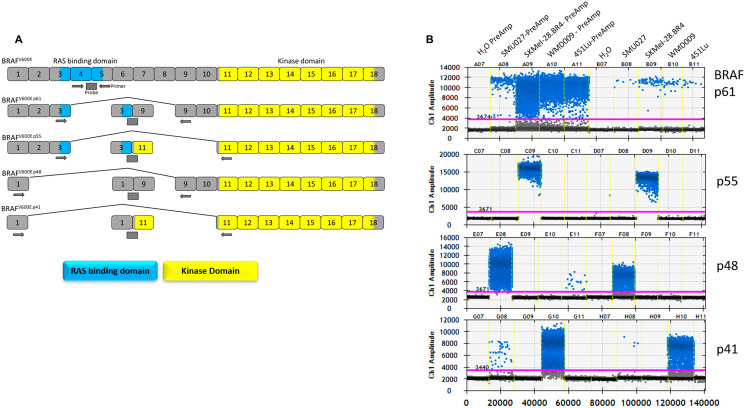
*BRAF* splice variants detection by droplet digital PCR. (**A**) *BRAF* exon organisation of the splicing variants and location of primers and probes used for detection of splicing variants. (**B**) Droplet digital PCR Ch1,1D plots of each *BRAF* splicing detection assay of melanoma cell lines carrying *BRAF* splicing variants, with and without the pre-amplification, (PreAmp) step.

Resistance to BRAF inhibitors usually involves MAPK reactivation, via mutations in *NRAS* or *MAP2K1/2*, upregulation of receptor tyrosine kinases, mutant *BRAF* gene amplification or alternative *BRAF* splicing [7–10]. In around 30% of resistant tumours, resistance to BRAF inhibition is conferred by alternative splicing via generation of BRAF isoforms lacking the RAS binding domain (RBD) encoded by exons 3–5 [7, 11]. In the absence of the RBD, these BRAF isoforms dimerise even in the presence of low levels of RAS, conferring and conferring resistance to BRAF inhibition [12]. Four *BRAF* splicing variants, referred to as p61, p55, p48 and p41 were named based on their predicted molecular weight (Figure 1A).

The analysis of tumour-derived biomarkers in blood of cancer patients is transforming clinical cancer pathology. Circulating tumour DNA (ctDNA) has emerged as a non-invasive biomarker to track tumour burden and allow monitoring of the cancer genome in blood across several malignancies including melanoma [13, 14]. We, and others, have previously demonstrated that increased melanoma ctDNA levels and the appearance of circulating mutations associated with of acquired resistance, provide highly specific, early information of relapse during BRAF targeted therapies [15, 16]. The latter supports a clinical model that incorporates the analysis of ctDNA in patients undergoing BRAF-inhibitor therapies as a routine test to detect early relapse and resistance [13, 14]. However, resistance involving aberrant splicing variants, cannot be detected through ctDNA analysis, and can only be identified using RNA.

Circulating tumour-derived cell free RNA or circulating tumour RNA (ctRNA) can be found in plasma from patients with cancer [17–19]. Various studies have also demonstrated the ability to amplify tumour-related mRNAs from sera of patients with melanoma, breast cancer, and other malignancies [20–24]. However, the possibility that extracellular RNA could survive in the blood has not been widely accepted, due to plasma containing potent ribonucleases capable of degrading cell-free RNA [25]. Regardless of this, studies have documented the presence of ctRNA in serum/plasma of cancer patients [26, 27].

Extracellular vesicles (EVs) are known to contain various RNA types, including microRNA, mRNA and circular RNA [28]. EV RNA has been described as having a substantial role in the transfer and movement of epigenetic information and is stable enough for whole transcriptome sequencing [29–31]. It is plausible that plasma cell-free RNA (cfRNA) is a mixture of RNA protected by RNA binding proteins and RNA contained within EVs. Here we explore whether alternative *BRAF* splicing variants can be detected in melanoma patients who have developed resistance to BRAF inhibitor therapy. We report a new methodological approach based on digital droplet polymerase chain reaction (ddPCR) to assess the presence of *BRAF* splicing variants in plasma cfRNA and EV-derived RNA. We interrogated plasma from 38 melanoma patients identified as having clinically diagnosed treatment resistance after initial response to BRAF inhibitor monotherapy or BRAF/MEK inhibitor combination. In addition, cfRNA was interrogated from an additional five patients who were confirmed to have tumour specific *BRAF* splicing variants in their progressing tumours. Finally, we evaluated the presence of *BRAF* splicing variants in EV RNA isolated from plasma of two melanoma patients failing BRAF inhibition.

## RESULTS

### Development of a sensitive assay for detection of *BRAF* splicing variants in plasma

We developed ddPCR assays for the detection of alternative *BRAF* splicing variants: p61, p55, p48 and p41, which are associated with resistance to BRAF inhibition, as well as the canonical full length *BRAF* (pFL) (Figure 1A). The assays were validated using three melanoma cell lines with resistance to BRAF inhibition and known to express alternative *BRAF* splicing variants (Figure 1B). As previously reported, SK-Mel-28. BR4 expresses BRAF p55 (exon 4-10Δ), SMU027 expresses BRAF p48 (exon 2-8Δ) and WMD009 expresses BRAF p41 (exon 2-10Δ) [9, 32]. Surprisingly without amplification all cell lines carry the BRAF p61, albeit at lower concentrations in comparison to the dominant splice variant expressed. Similarly, SMU027 expressed low levels of BRAF p41 compared to WMD009.

To enhance sensitivity of detection of *BRAF* splicing variants, a pre-amplification step was introduced. This resulted in a significant enhancement of the signal without loss of specificity (Figure 1B). Importantly, the introduced pre-amplification step maintained a linear relation between copies of input cDNA and output *BRAF* splicing variant copies (R^2^ = 0.9986, *p* < 0.0001), with a 6,396 ± 569 fold increase in *BRAF* splicing variant copies (Supplementary Figure 2).

We tested plasma from nine healthy volunteers, to confirm that the *BRAF* splicing variants are not commonly found in normal plasma and that the assay does not introduce artefacts or induce false detection of BRAF alternative splicing variants (Supplementary Figure 3).

### Analysis of *BRAF* splicing variants in plasma of melanoma patients

**Figure 2:**
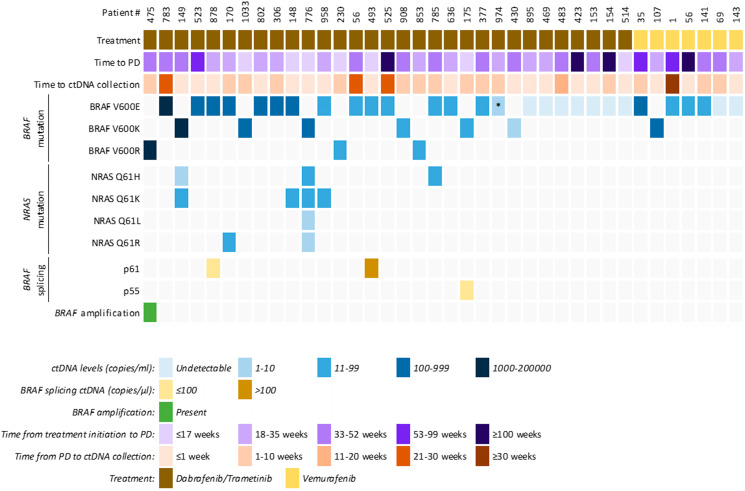
Clinical and ctDNA profile of patients that showed clinical progressive disease to targeted therapy. Overview of ctDNA results (copies/mL plasma) from *BRAF*, *NRAS*, *BRAF* splicing and *BRAF* amplification in 38 melanoma patients. Each column is an individual patient, showing their clinical characteristics, quantitative ctDNA results (copies/ml or μl of plasma) and presence of *BRAF* amplification. ^*^ *BRAF* V600E c.1799_1800GT>AA, p. (Val600Glu), also known as *BRAF* V600E2 mutation.

We prospectively collected longitudinal plasma samples from 38 patients: 7 vemurafenib and 31 dabrafenib/trametinib treated cases (Supplementary Figure 4). Of the 7 vemurafenib cases, 2 had baseline plasma for ctDNA analysis, and of these 1 experienced a decrease in mutant *BRAF* during treatment (MM143), while the other had an increase in mutant *BRAF* copies during treatment (MM141). Of the 31 dabrafenib/trametinib cases, 25 had assessable baseline plasma, and of these 24 cases had detectable ctDNA (as mutant *BRAF* copies) at baseline. In 19 of the 24 cases, ctDNA decreased during treatment in comparison to the baseline sample. Analysis of blood samples collected at the time of progressive disease (red points – Supplementary Figure 4), identified 29 patients (5 on vemurafenib and 24 on combination dabrafenib/trametinib therapy) with detectable ctDNA at progression. Of those, seven had additional *NRAS* Q61 mutations and one had a *BRAF* gene amplification detectable in plasma (Figure 2 and Supplementary Table 1).

**Figure 3:**
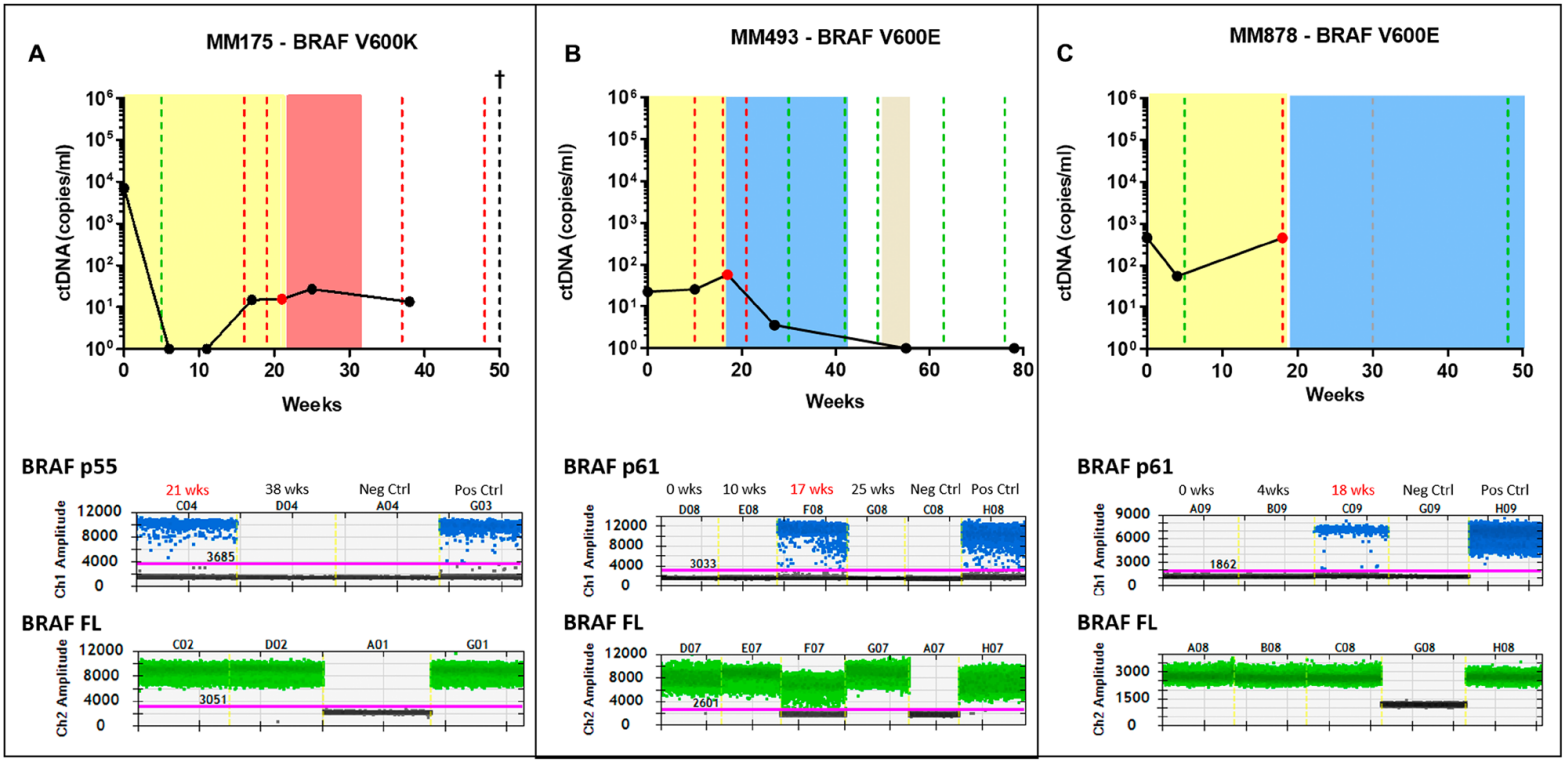
Detection of splice variants in the combination therapy cohort. Three patients undergoing combination BRAFi therapy were positive for a splicing variant. Patients MM175 (**A**) and patients MM493 (**B**), were both positive for the p61 variant at the point of progressive disease shown in the droplet digital plots. Patient MM878 (**C**), was positive for the p55 variant also shown in the droplet digital plot. The longitudinal graphs represent the ctDNA tracking of the activating mutation for the patients. Red, green and grey dotted lines represent clinical response, progressive disease and stable disease respectively. The singular red dot represents the time-initial point tested positive for the splicing variants and neighbouring time-points were tested to confirm the presence or absence of the splicing variants. The coloured area indicates the period during which systemic therapy was administered; colours representing dabrafenib/trametinib (yellow), ipilimumab (red) and pembrolizumab (blue). Droplet digital plot represents the positivity for the indicated BRAF splicing variant (blue dots). BRAF full length (BRAF FL, green dots) was use as positive control for RNA extraction and amplification. No template control and positive control (SK-Mel-28. BR4) were included in each test.

Three patients (MM175, MM493 and MM878) had detectable *BRAF* splicing variants in plasma using our assay, indicating the presence of ctRNA. *BRAF* splicing variants were not detected in the pre-treatment plasma (MM493, MM878) despite clear detectable ctDNA (Figure 3). Unfortunately, no plasma collected at baseline was available from MM175 for *BRAF* splicing variant assessment.

MM175 presented with multiple spine, rib and liver metastases. The patient received dabrafenib/trametinib combination treatment and showed a partial response at 5 weeks, with undetectable ctDNA (Figure 3A). Subsequent scans confirmed progression with the patient being moved onto ipilimumab immunotherapy. Analysis of plasma ctRNA at the final point of dabrafenib/trametinib progression (21 weeks) and after change of therapy to ipilimumab (38 weeks), revealed that the *BRAF* splicing variant p55 was present only at the time of progression but undetectable while on ipilimumab treatment, despite having sustained *BRAF* V600K ctDNA levels.

MM493 presented with multiple brain, lung and abdomen metastases. One brain metastasis was resected while and the others were treated with whole brain radiotherapy. This patient received dabrafenib/trametinib combination treatment with partial response at 8 weeks and limited decrease in ctDNA (Figure 3B). BRAF p61, but no other *BRAF* splicing variants, was detected at the time of progression (17 weeks, Figure 3B). Plasma collected at baseline, during response (10 weeks) or after starting pembrolizumab (25 weeks) did not have detectable *BRAF* splicing variants. *BRAF* V600E ctDNA was detectable in this patient at all four-time points analysed but became undetectable by week 54 in response to treatment with pembrolizumab.

The third patient (MM878) presented with widespread melanoma in lung, lymph node, adrenal, bone and gallbladder. The patient was placed on dabrafenib/trametinib combination treatment and a partial metabolic response was observed 5 weeks later which was concordant with a decrease from 458 copies/ml to 56 copies/ml decrease in *BRAF* V600E ctDNA (Figure 3C). At the time of progression additional skeletal metastases were observed by PET scan 18 weeks into therapy, with ctDNA levels significantly increased to 458 copies/ml, with the PET scan confirming progression of several skeletal metastases. The BRAF p61 variant was detectable in plasma at the point of progression, but was undetectable at baseline or the first follow up sample collected 4 weeks into therapy (Figure 3C).

**Figure 4:**
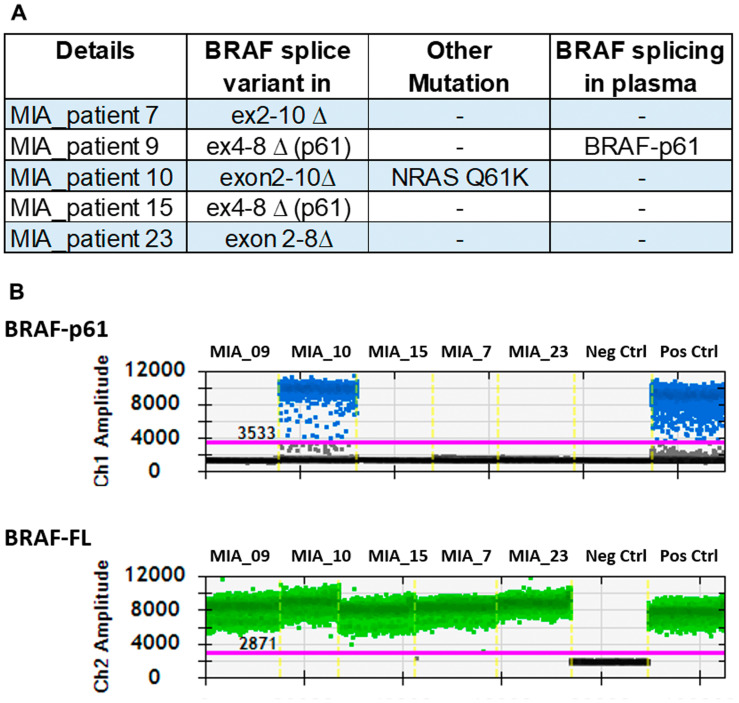
Detection of *BRAF* splice variants in patients known to have the splice variant. (**A**) Patients who had confirmation of splicing variants in the tumour were investigated to confirm the presence of the variant in the plasma. (**B**) The droplet digital plot represents the positivity for p61 in one of the samples.

To validate our findings, we next tested the plasma derived from five melanoma patients that were treated with vemurafenib monotherapy who developed resistance. Tumour samples obtained at the time of progression had confirmed *BRAF* splicing variants by transcriptome analysis [8] (Figure 4). We analysed plasma samples from these patients collected near the time of resecting the progressed tumour. Only one of these five cases (MIA_10) had a detectable splicing variant (BRAF p61) in plasma which was concordant with the results of the matched tumour sample. *BRAF* splicing variants were not detectable in the baseline plasmas from theses patient.

### *BRAF* splicing variants are associated with extracellular vesicles

Plasma extracellular RNAs are thought to be carried by extracellular vesicles [33]. To evaluate whether *BRAF* splicing variants are found in EVs, we analysed extracellular vesicles extracted from the plasma of patients MM493 and MM878. EVs isolated from the supernatant of SK-Mel-28. BR4 cell line and from the plasma of a healthy control were used as positive and negative controls, respectively.

**Figure 5:**
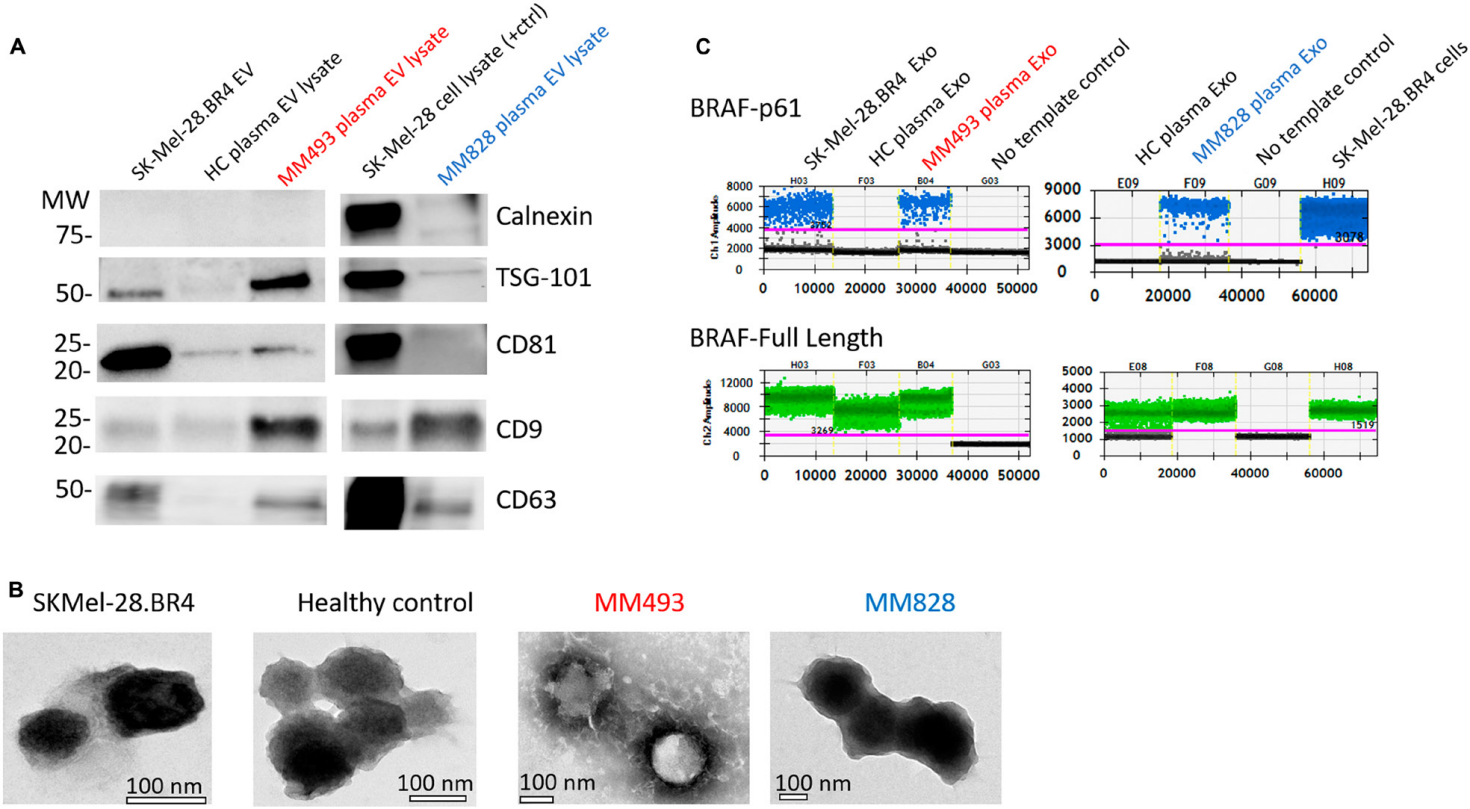
Validation of EV isolation from exo-easy and confirmation of splicing variant presence. (**A**) EVs isolated using the ExoEasy Kit were tested for the associated markers TSG-101, CD81, CD9 and CD63 and the negative marker Calnexin. Loading was standardised at 4 μg across all EVs preparations. (**B**) Electron micrographs were used to determine the presence of EV/exosomes in the appropriate size range. (**C**) Droplet digital testing for BRAF p61 (blue dots) and FL-BRAF (green dots) in EV-derived RNA from the patients, healthy controls and cell line SK-Mel-28. BR4.

The effective isolation of EVs was confirmed by western blot analysis, with positive signal for the tetraspanins CD9, CD63, CD81 and Tumour Susceptibility Gene 101 (TSG101), while negative for the cellular marker calnexin (Figure 5A and Supplementary Figure 5). Transmission electron microscopy (TEM) was used to visualise the extracted EVs, demonstrating the presence of exosomes with a size of 100 nm (Figure 5B). Analysis of EV RNA from SK-Mel-28. BR4 cells confirmed the presence of BRAF p61 and BRAF FL, while EV RNA from healthy volunteer only carried BRAF FL variants. EV RNA from both MM493 and MM878 contained BRAF p61 transcripts (Figure 5C).

## DISCUSSION

The emergence of *BRAF* splicing variants is a common mechanism of escape to BRAF inhibition in melanoma [10, 12]. Elucidation of the molecular escape mechanisms usually requires the sourcing of tumour tissue at the time of progressive disease. Here we show that tumour specific splicing variants, known to mediate treatment resistance, can be detected in plasma.

Our study demonstrated the detection of resistance mediating *BRAF* splicing variants in ctRNA of melanoma patients failing BRAF inhibiting therapies. Moreover, we showed that these *BRAF* splicing variants are co-purified or associated with EVs.

The frequency at which we detect splicing variants in this cohort was low. Tumour analyses within cohorts treated with BRAF inhibiting monotherapies suggest a prevalence of approximately 30% for splicing variants [8, 10]. Long *et al.* suggested that *BRAF* splicing were unlikely to occur in combination therapy as MAPK signalling driven by such variants will require MEK activity and therefore will remain sensitive to MEK inhibitors [9, 34]. Nevertheless, we did not detect *BRAF* splicing variants in any of the 7 patients treated with vemurafenib monotherapy. In contrast, we observed *BRAF* splicing variants in 3 of 31 patients treated with combination BRAF/MEK inhibitors. A study by Wagle et al., reported the detection of BRAF p41 in the post-treatment tumour of 1 out of 5 patients that developed resistance to dabrafenib/trametinib [35].

In this study, we failed to detect *BRAF* splicing variants in 4 of 5 patients for which the tumours were confirmed to carry *BRAF* splicing variants by tissue analysis. The isolation and presence of circulating RNA was confirmed in all samples through the presence of BRAF full length. Moreover, we showed that our method had high analytical sensitivity and specificity. However, several technical and biological reasons may explain the low clinical sensitivity of the test. Similar to ctDNA, ctRNA detection is likely to be affected by tumour burden, metabolic activity and location of the tumour [36, 37]. In addition, pre-analytical conditions have not been optimised for the preservation and extraction of ctRNA or EV-derived mRNA in the same way that it has been done for ctDNA [38].

The presence of ctRNA has been previously reported in plasma and other body fluids of cancer patients [17, 39–41]. Early reports published by Lo *et al.* [42] indicated the presence of tumour derived ctRNA in the plasma of nasopharyngeal carcinoma patients. Beyond this, ctRNA analysis has been expanded primarily into cancer detection cancer or monitoring response [43]. For example, plasma ctRNA expression of the carcinoembryonic antigen has been used for diagnosis of early stage prostate cancer [43] and plasma *TERT* mRNA levels have demonstrated to be predictive of response to chemoradiotherapy in rectal cancers [44]. Thus, ctRNA shows promise as an additional monitoring tool that can be used in combination with ctDNA and conventional methods. The advantage of ctRNA is implied through the assessment of transcriptional activity of genes.

The effective isolation of EVs was validated using western blot and transmission electron microscopy. Purity was confirmed through negativity of calnexin, along with the positivity of CD9, CD63, CD81 or TSG101 which are EV associated proteins [45]. Patient MM828-EV sample was CD81 negative but positive for the other markers, demonstrating that reliance on one or two markers poses a risk of false negativity. The heterogeneity of EV-associated proteins has been reported previously [46–48], suggesting that a panel of markers is required to validate the presence of EVs.

EVs have been established as carriers of various types of RNA, including mRNA, miRNA and long non-coding RNAs [49–51]. The EV RNA from the patients MM493 and MM878 were found positive for BRAF-p61, matching the results obtained via their plasma ctRNA. This suggests that ctRNA may be carried totally or partially by EVs, and that these vesicles may be protecting RNA from degradation [49, 52, 53].

Alterations on splicing variants have been shown to be associated with cancer progression and treatment resistance [54]. Splicing variants of the androgen receptor, in particular AR-V7, have been found to be significantly higher in hormone refractory prostate cancer and is associated with poor clinical outcomes [55]. Similar to BRAF-splicing variants, AR-V7 can be detected in plasma EV RNA [53, 56]. Altogether, this supports an alternative liquid biopsy modality based on EV RNA or ctRNA.

Liquid biopsies have emerged as a non-invasive approach to assess disease burden and the genetic evolution of tumours in response to therapy [15, 57–59]. Our study provides a foundation for the detection of splicing variants in both ctRNA and EV RNA in melanoma patients failing MAPK inhibition, although its clinical validity requires further evaluation in independent cohorts. This work constitutes an important proof of concept that in addition to plasma ctDNA, ctRNA can provide important tumour specific information related to the development of resistance. However pre-analytical conditions for ctRNA analysis still require optimisation to enable its clinical application.

## MATERIALS AND METHODS

### Patient selection

A total of 38 participants with BRAF mutant metastatic melanoma were treated with vemurafenib or with dabrafenib and trametinib combination as per approved label were enrolled (Supplementary Table 1). Patients were required to have a recorded objective response to therapy, either partial or complete confirmed by CT or PET scans. All patients gave their signed informed consent before blood collection and data analysis. The study was performed in accordance with the Declaration of Helsinki and study protocols were approved by the Human Ethics Committees at Edith Cowan University (No.11543) and Sir Charles Gardner Hospital (No.2013-246).

Plasma from another five patients treated recruited at the Melanoma Institute Australia and affiliated hospitals were included in this study (Melanoma Institute Australia Biospecimen Bank for Melanoma Research ×11-0289 & HREC/11/RPAH/444 approved through Sydney Local Health District). These patients were selected as they were previously identified as to have *BRAF* splicing variants in their progressing tumour after failing treatment with dabrafenib monotherapy.

### Cell culture

Melanoma cell line 451Lu was obtained from the Wistar Institute. SK-Mel-28. BR4, SMU027 and WMD009 were previously reported to carry *BRAF* splicing variants [9, 32]. All cell lines were cultured in Dulbecco’s Modified Eagle Medium (DMEM) (Thermo Fisher Scientific, Carlsbad, CA, USA) supplemented with 10% Foetal Bovine Serum (Thermo Fisher Scientific, Carlsbad, CA, USA) at 37°C and 5% CO_2_.

### RNA extraction from cell lines

RNA was extracted using the All Prep DNA/RNA Mini kit (Qiagen, Hilden, Germany) according to the manufacturer’s instructions. RNA was eluted in RNAse free water and stored at –80°C until analysis.

### Circulating nucleic acid extraction

Blood samples were collected into EDTA vacutainer tubes and plasma was separated within 24 hours by centrifugation at 300 g for 20 minutes, followed by a second centrifugation at 4700 g for 10 minutes and then stored at –80°C until extraction. Plasma cell-free nucleic acids (cfNA) were isolated from healthy donors and AJCC stage IV metastatic melanoma patients using the QIAamp Circulating Nucleic Acid Kit (Qiagen, Hilden, Germany) as per the manufacturer’s instructions. cfNA was eluted in 40 μl AVE buffer (Qiagen, Hilden, Germany) and stored at –80°C.

### Reverse transcription and preamplification

Extracted RNA (5 μl) from all samples were used for cDNA preparation using the SuperScript^®^ VILO™ cDNA Synthesis Kit (Thermo Fisher Scientific, Carlsbad, CA, USA) followed by a preamplification using the TaqMan^®^ PreAmp Master Mix Kit (Thermo Fisher Scientific, Carlsbad, CA, USA) and all the primers for the *BRAF* splicing variants at 180 nM, following manufacturer cycling conditions (95°C for 10 minutes, followed by 14 cycles of 95°C for 15 seconds and 60°C for 4 minutes, with a final inactivation step at 99°C for 10 minutes). Linearity of the pre-amplification step was assessed by linear regression of a serial dilution curve (Supplementary Figure 2).

### Droplet digital PCR

Primer pairs were designed to amplify over the unique exon-exon junction of each splicing variant, with probes overlapping the junction (Figure 1A and Supplementary Table 2). Probes were custom synthesised by Integrated DNA Technologies (IDT, San Jose, CA, USA). For ctDNA analysis, samples were analysed for the presence of *BRAF* or *NRAS* mutant DNA as described previously [15, 60].

Amplifications were performed in a 20 μL reaction containing 1× ddPCR Supermix for Probes (No dUTP, Bio-Rad), 1× Q solution (Qiagen, Hilden, Germany), 250 nM of each probe and 900 nM of each primer plus template. Droplets were generated using the automatic droplet generator QX200 AutoDG (Bio-Rad, Hercules, CA, USA). Amplifications were performed using the following cycling conditions: 1 cycle of 95°C (2.5C/s ramp) for 10 minutes, 40 cycles of 94°C (2.5C/s ramp) for 30 seconds and 55°C for 1 minute, followed by 1 cycle of 98°C (2.5C/s ramp) for 10 minutes. Droplets were analysed through a QX200 droplet reader (Bio-Rad, Hercules, CA, USA). QuantaSoft analysis software (Bio-Rad, Hercules, CA, USA) was used to acquire and analyse data.

### BRAF amplification analysis

We optimised a ddPCR assay to assess the presence of *BRAF* amplifications using plasma-derived cfDNA. *BRAF* copy number was assessed relative to a reference gene situated in the centromere of chromosome 7 (*VOPP1* gene, 7p11.2). For the assessment of BRAF: VOPP1 ratio, a ddPCR assay was performed with the Bio-Rad QX200 system using custom primers/probe sets against *BRAF* [60] and *VOPP1* (Bio-Rad, Hercules, CA, USA). The *BRAF: VOPP1* concentration ratio was determined by dividing the *BRAF* concentration by the *VOPP1* concentration offered by the QuantaSoft software (Bio-Rad, Hercules, CA, USA).

We first determined the optimal threshold to define an elevated plasma DNA ddPCR BRAF: VOPP1 ratio using a standard curve of genomic DNA extracted from healthy volunteer WBC combined with different percentages of genomic DNA extracted from a melanoma cell line with known *BRAF* amplification (Supplementary Figure 1). Plasma-derived cfDNA from six healthy volunteers were used to evaluate specificity. Patient samples with mutant *BRAF* frequency abundance of 3% or higher were tested for *BRAF* amplification.

### Extracellular vesicle isolation

For EVs isolation, cell lines at 50% confluence were washed three times with PBS and cultured in DMEM supplemented with exosome-depleted FBS (Thermo Fisher Scientific, Carlsbad, CA, USA) for 18 hours at 37°C and 5% CO2. Following this, cell culture media was harvested and centrifuged at 300 g for 5 minutes at 4°C. The supernatant was then transferred to a clean tube and re-centrifuged at 2000 g for 20 minutes. Cleared supernatant was used for EV isolation using the ExoEasy kit (Qiagen, Hilden, Germany) following manufacturer’s instructions. EVs were also isolated from 4 ml of plasma from melanoma patients and heathy volunteers using the ExoEasy kit (Qiagen) RNA was isolated from the extracted EVs using the RNeasy Micro Kit (Qiagen, Hilden, Germany) following manufacturer’s instructions. RNA was eluted in 14 μL.

### Western blots

SK-Mel-28 cell lysate was prepared to be used as positive control. Cells were harvested using trypsin EDTA (Thermo Fisher Scientific, Carlsbad, CA, USA). Cell viability was then determined using trypan blue. Cells were then pelleted at 300 g for five minutes, washed twice in PBS and lysed with radioimmunoprecipitation (RIPA) assay buffer (Sigma-Aldrich, St. Louis, Missouri) containing a complete protease inhibitor tablet (Roche, Basel, Switzerland). Cell lysates were incubated for 30 minutes on ice prior to being cleared with a 13,000 g spin for five minutes, with the supernatant being transferred into a clean tube and stored at –20°C until use.

EV pellets were resuspended in PBS prior to mixing 1:1 with RIPA (Sigma-Aldrich, St. Louis, Missouri) supplemented with protease inhibitor cocktail (Roche, Basel, Switzerland). EV lysates were incubated for 30 minutes on ice and then stored at –20°C until use. Extracted EV proteins were diluted to 1:4 in Lamelli Buffer (Bio-Rad, Australia), incubated at 95°C for 5 minutes and resolved on a mini TGX 8–16% stain free gel (Bio-Rad). Proteins were transferred onto a nitrocellulose blotting membrane using the Trans-Blot^®^ Turbo™ Transfer System at a constant voltage of 25V for seven minutes (Bio-Rad, Australia). The membranes were blocked in 5% milk tris buffered saline (TBS) for one hour at room temperature before being probed with primary antibody TSG101 (1:1000, clone EPR7130B, Abcam), CD9 (1:500, clone MM2/57, Life-Technologies), CD81 (1:500, clone 1.3.3.22, Life-Technologies). CD63 (1:1000, clone H5C6, BD-Biosciences, calnexin (1:500, clone AF18, Life-Technologies) diluted in 0.5% milk TBS 0.05% Tween 20. The membrane was washed three times for ten minutes with TBS 0.05% tween and subsequently probed with secondary antibodies (sheep anti-mouse IgG-HRP conjugate, polyclonal, 1:2000, GE Healthcare, donkey anti-rabbit IgG-HRP conjugate, polyclonal, 1:2000, GE Healthcare) diluted in 0.5% milk TBS .05% Tween 20. Signals were detected with the GE healthcare Amersham™ ECL™ reagent and were subsequently imaged using the ChemiDoc™ Touch Imaging System (Bio-Rad, Hercules, CA, USA). Images were processed using Image Lab™ software v6.0 (Bio-Rad, Hercules, CA, USA).

### Transmission electron microscopy

EVs were resuspended in PBS and fixed in 2% paraformaldehyde prior to transfer onto 200 mesh Formvar-carbon coated copper grids (ProSciTech, Kirwan, QLD). EVs were adsorbed for 15 minutes at room temperature prior to being washed four times in filtered H_2_O. Samples were then fixed in 1% glutaraldehyde and contrasted with 1% uranyl acetate for 2 minutes before being left for 20 minutes to dry at room temperature. EVs were visualised using JEOL JEM-2100 electron microscope (JEOL, Tokyo, Japan) at an operating voltage of 80 kV. Images were captured using an 11M pixel Gatan Orius digital camera (Gatan, Pleasanton, CA, USA).

## SUPPLEMENTARY MATERIALS




