# Supplementary material for: Detection of BRAF splicing variants in plasma-derived cell-free nucleic acids and extracellular vesicles of melanoma patients failing targeted therapy therapies
Source: Oncotarget. 2020 Nov 3;11(44):4016–27. doi: 10.18632/oncotarget.27790 (PMC7646833; doi:10.18632/oncotarget.27790)
Supplement: Supplementary file 1 [file oncotarget-11-4016-s001.pdf]

Detection of *BRAF* splicing variants in plasma-derived cell-free nucleic acids and extracellular vesicles of melanoma patients failing targeted therapy therapies

SUPPLEMENTARY MATERIALS

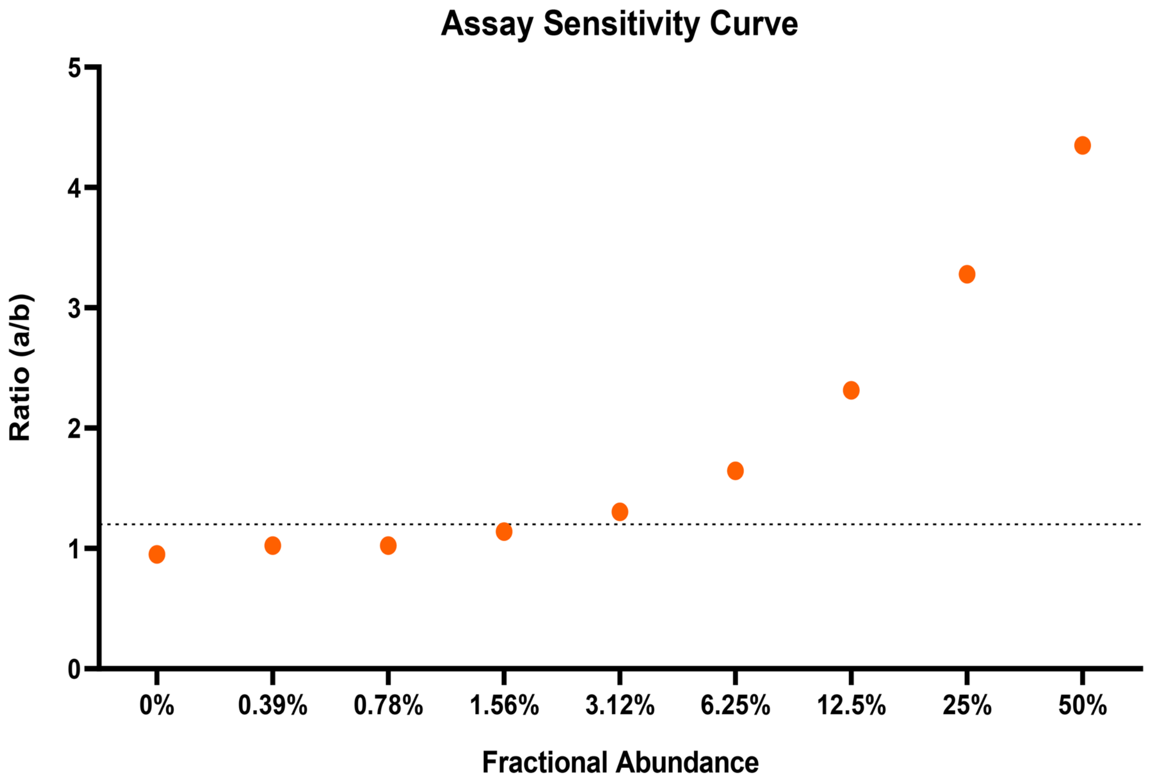

Supplementary Figure 1: Sensitivity curve of the *BRAF* amplification assay. Relationship of ratio and variable fractional abundance of cell line mixtures. Clinical samples with ≥ 3% *BRAF* mutant FA were tested for *BRAF* amplification.

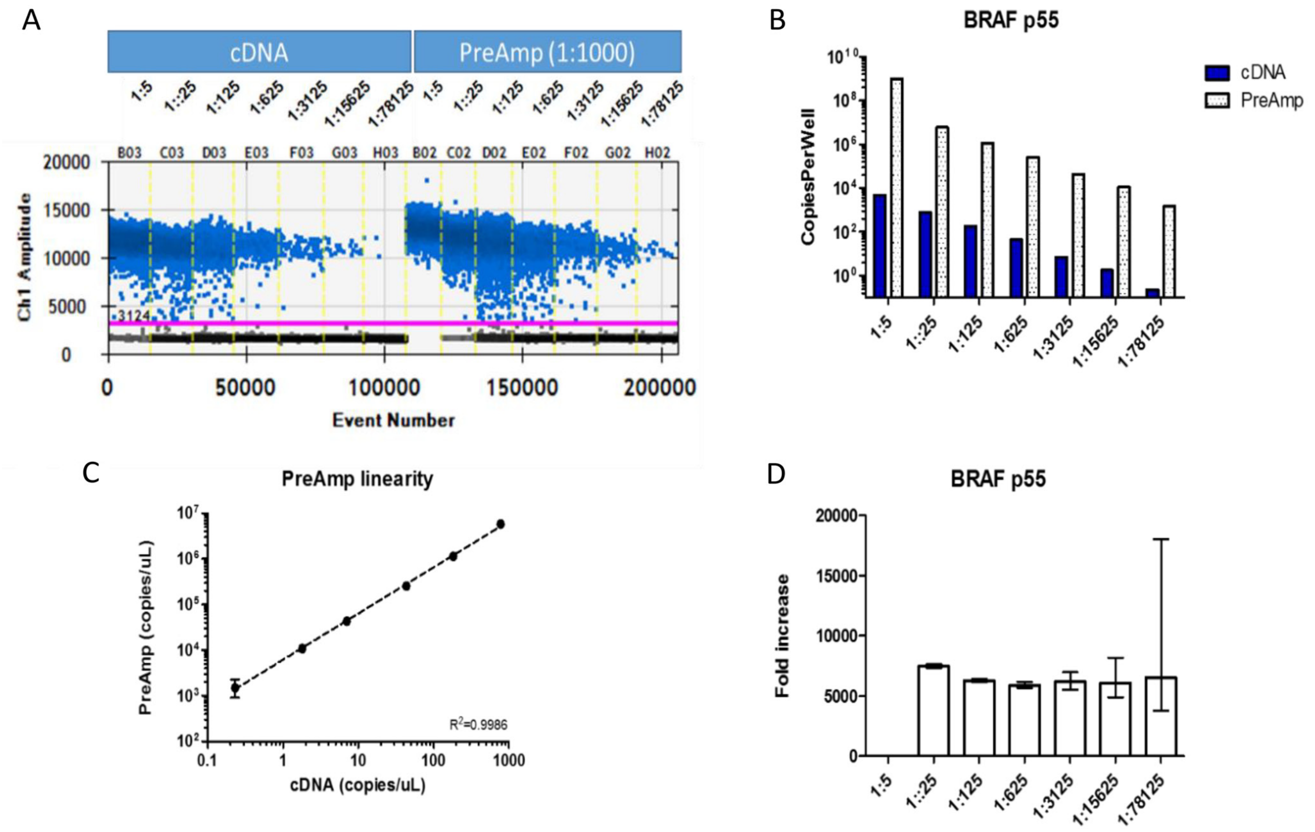

Supplementary Figure 2: Linearity of *BRAF* pre-amplification. (**A**) 1D plots of ddPCR specific for BRAFp55 with increasing cDNA input with and without prior pre-amplification. (**B**) Copies per well output from ddPCR. The error bars indicate the upper and lower limit of Poisson each result. (**C**) Linear regression of cDNA copies prior to and after pre-amplification. The R^2 value of the analysis is indicated. Error bars of three replicates are indicated for each dilution. (**D**) Fold change for each one of the concentrations, with error bars for each dilution indicated.

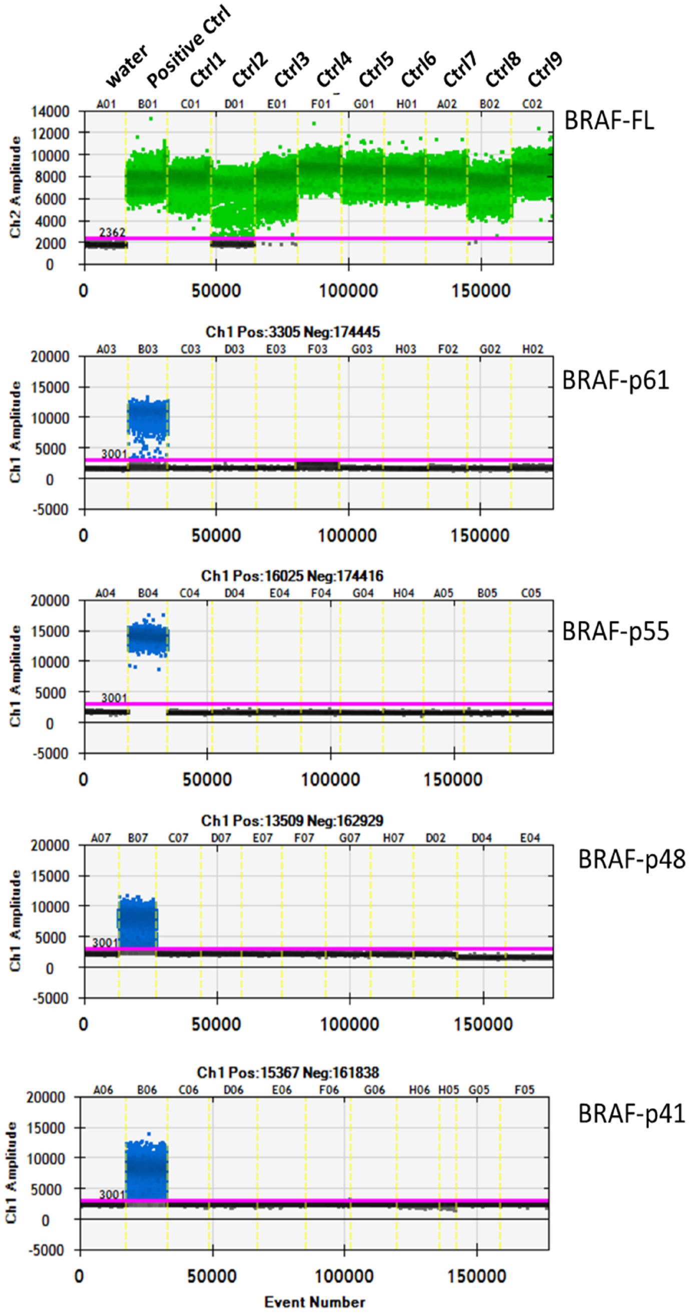

	Sex	Age
Ctrl 1	Male	54
Ctrl 2	Male	41
Ctrl 3	Female	48
Ctrl 4	Female	47
Ctrl 5	Female	48
Ctrl 6	Female	41
Ctrl 7	Female	52
Ctrl 8	Male	58
Ctrl 9	Male	51

Supplementary Figure 3: Specificity of *BRAF* splicing variants detection in plasma. Cell free nucleic acid isolated from plasma of 9 healthy individuals were tested for all four *BRAF* splicing variants and full length BRAF. Only full length BRAF (green) was detected in all 9 healthy control samples. Age and sex of all the donors are specified in the inserted table. Positive control constitutes a mix of RNA from cell lines expressing the corresponding variant as sown in Figure 1B: SK-Mel-28.BR4 (p61 and p55), SMU027 (p48) and WMD009 (p41).

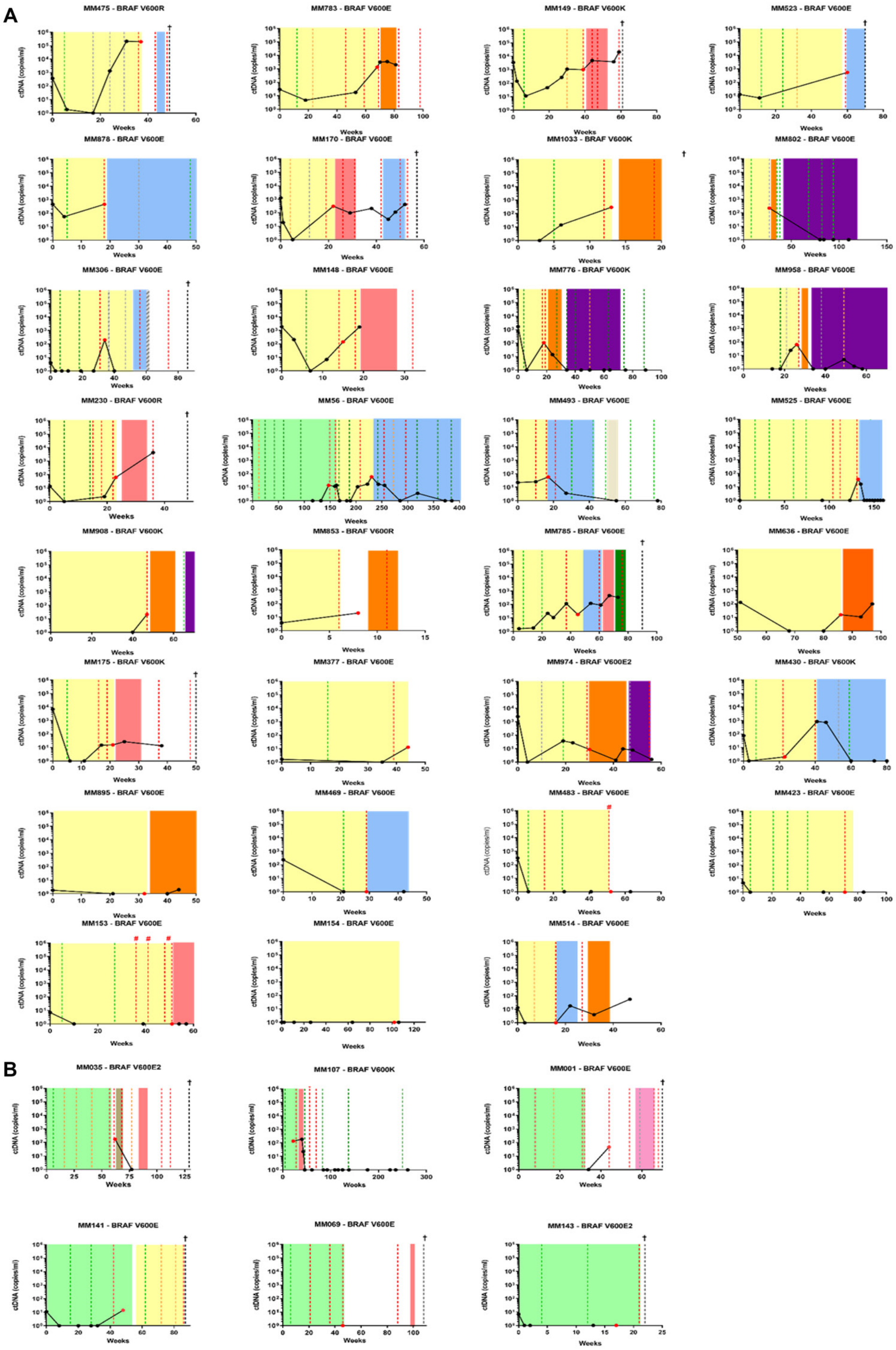

Supplementary Figure 4: Monitoring ctDNA levels during clinical disease course. (**A** and **B**) Plasma ctDNA concentrations for patients that progressed on dabrafenib/trametinib (A) or vemurafenib (B). Radiological outcomes such as, response, stable disease, mixed response and progressive disease are represented by a dashed green, orange, gray, or red line, respectively. A black dashed line represents time of death and indicated with a black cross (†). The coloured area indicates the period during which systemic therapy was administered; colours representing dabrafenib/trametinib, nivolumab, ipilimumab, vemurafenib, pembrolizumab, ipilimumab/nivolumab, vemurafenib/cobimetinib, dabrafenib, DTIC, bevacizumab. [#]Intracranial disease progression with no extracranial involvement.

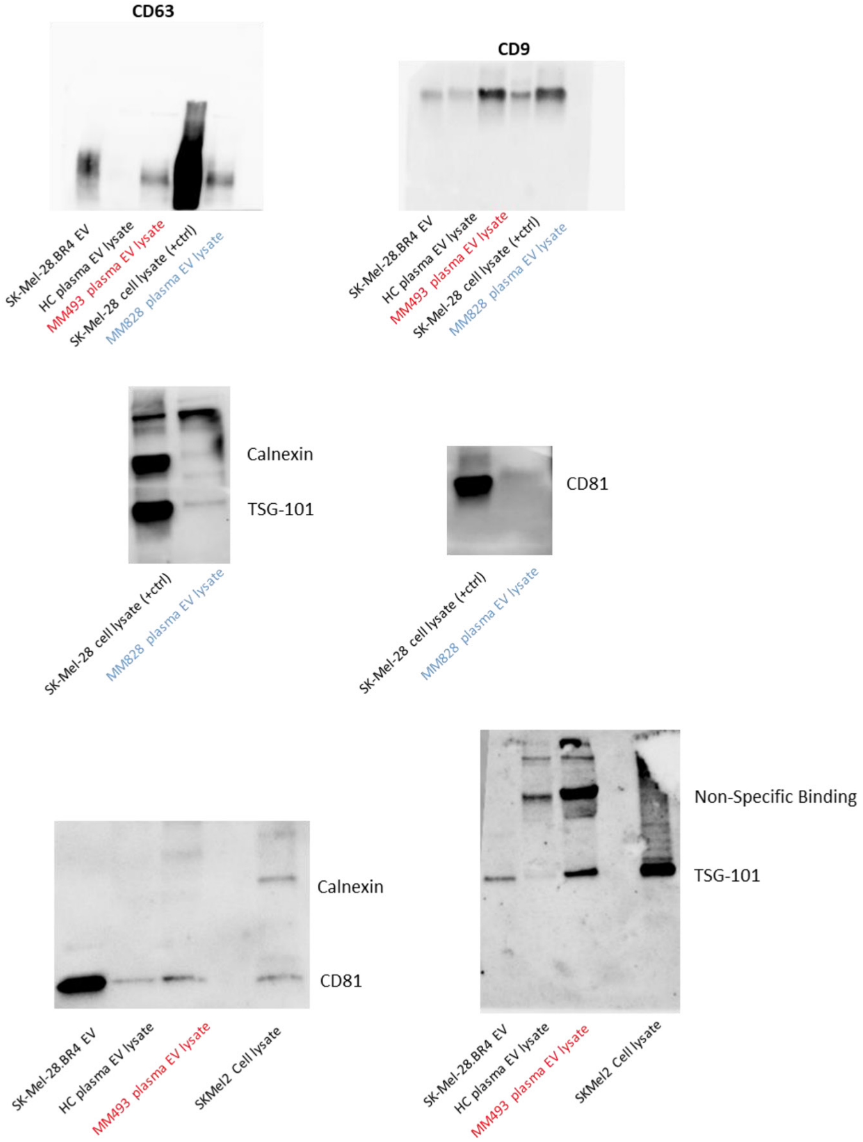

Supplementary Figure 5: Uncropped images from Western blots shown in Figure 5A.

Supplementary Table 1: Progressive disease samples analysed for the presence of BRAF splicing.
See Supplementary Table 1

Supplementary Table 2: Primers and probes for detection of BRAF splicing variants

Name	Sequence	Length (bp)	Tm (˚C)
BRAF-FL-Forward	AATTGCATGTGGAAGTGTTG	20	60.97
BRAF-FL-Reverse	GCTTTCGACAAAAGTCACAA	20	60.75
BRAF-p61-Forward	TCACCACAAAAACCTATCGT	20	61.03
BRAF-p61-Reverse	CACGAAATCCTTGGTCTCTA	20	60.74
BRAF-p55–Reverse	AATCTGCCCATCAGGAAT	18	60.23
BRAF-p48-Forward	TTCAACGGGGACATGGA	17	62.77
BRAF-FL	/5-HEX/CACACAACT/ZEN/TTGTACGAA/3IABkFQ/	18	56.71
BRAF-p61	/56-FAM/AGGACAGTG/ZEN/GACTTGATT/3IABkFQ/	18	59.6
BRAF-p55	/56-FAM/TACCAAGTG/ZEN/TTTTCACTGTCCTC/3IABkFQ/	23	64.06
BRAF-p48	/56-FAM/ATCAAGTCC/ZEN/TCCTCCGGA/3IABkFQ/	18	64.1
BRAF-p41	/56-FAM/CCAAGTGTT/ZEN/TTCTCCTCCGG/3IABkFQ/	20	64.97
