# Supplementary material for: Detection of BRAF splicing variants in plasma-derived cell-free nucleic acids and extracellular vesicles of melanoma patients failing targeted therapy therapies
Source: Oncotarget. 2020 Nov 3;11(44):4016–27. doi: 10.18632/oncotarget.27790 (PMC7646833; doi:10.18632/oncotarget.27790)
Supplement: Supplementary file 2 [file oncotarget-11-4016-s002.docx]

| **Patient ID** | **Date of Collection** | ***BRAF* mutation** | **ctDNA (copies/ml)** | **Fractional abundance** | ***NRAS* mutation** | **ctDNA (copies/ml)** | **Fractional abundance** | ***BRAF amplification*** | ***BRAF* Splicing** | ***BRAF* splicing (copies/µl)** | |
| --- | --- | --- | --- | --- | --- | --- | --- | --- | --- | --- | --- |
| ***Combination therapy (Dabrafenib/Trametinib)*** | | | | | | | | | | |  |
| MM475 | 2/11/2016 | BRAF V600R | 196800.0 | 96.3 | Neg |  |  | 6.6 | - |  |  |
| MM783 | 29/03/2019 | BRAF V600E | 1380.0 | 28.8 | Neg |  |  | - | - |  |  |
| MM149 | 1/04/2014 | BRAF V600K | 1020.0 | 11.5 | Q61R | 67.5/9.0 | 0.66/0.03 | nt | - |  |  |
| MM523 | 26/09/2017 | BRAF V600E | 556.0 | 19.7 | Neg |  |  | - | - |  |  |
| MM878 | 10/10/2018 | BRAF V600E | 458.0 | 13.1 | Neg |  |  | **-** | **BRAF p61** | 21.5 |  |
| MM170 | 19/03/2014 | BRAF V600E | 308.0 | 14.8 | Q61R | 30.0 | 1.2 | - | - |  |  |
| MM1033 | 30/01/2019 | BRAF V600K | 292.0 | 4.2 | Neg |  |  | - | - |  |  |
| MM802 | 23/01/2018 | BRAF V600E | 222.0 | 12.4 | Q61R |  |  | nt | - |  |  |
| MM306 | 21/09/2015 | BRAF V600E | 202.0 | 3.1 | Neg | 2.8 | 0.0 | - | - |  |  |
| MM148 | 22/10/2013 | BRAF V600E | 143.0 | 5.3 | Q61K | 62.3 | 1.8 | nt | - |  |  |
| MM776 | 27/03/2018 | BRAF V600K | 106.0 | 5.3 | Q61H/K/L/R |  |  | - | - |  |  |
| MM958 | 3/12/2018 | BRAF V600E | 64.0 | 6.4 | Q61K | 30.0 | 2.0 | - | - |  |  |
| MM230 | 4/11/2014 | BRAF V600R | 62.0 | 0.8 | Neg |  |  | nt | - |  |  |
| MM056 | 15/06/2016 | BRAF V600E | 60.0 | 4.4 | Neg |  |  | - | - |  |  |
| MM493 | 3/08/2016 | BRAF V600E | 58.0 | 1.0 | Neg |  |  | nt | **BRAF p61** | 153.0 |  |
| MM525 | 14/02/2019 | BRAF V600E | 36.7 | 0.6 | Neg |  |  | nt | - |  |  |
| MM908 | 20/09/2018 | BRAF V600K | 21.1 | 2.2 | Neg |  |  | nt | - |  |  |
| MM853 | 18/07/2018 | BRAF V600R | 20.0 | 0.3 | Neg |  |  | nt | - |  |  |
| MM785 | 21/09/2018 | BRAF V600E | 18.0 | 0.6 | Q61H | 54.0 | 1.7 | nt | - |  |  |
| MM636 | 14/02/2018 | BRAF V600E | 16.0 | 1.2 | Neg |  |  | nt | - |  |  |
| MM175 | 19/03/2014 | BRAF V600K | 15.8 | 0.8 | Neg |  |  | nt | **BRAF p55** | 53.5 |  |
| MM377 | 13/05/2016 | BRAF V600E | 12.8 | 0.1 | Neg |  |  | nt | - |  |  |
| MM974 | 12/04/2019 | BRAF V600E2 | 8.8 | 0.2 | Neg |  |  | nt | - |  |  |
| MM430 | 30/03/2016 | BRAF V600K | 1.8 | 0.5 | Neg |  |  | nt | - |  |  |
| MM895 | 20/02/2019 | BRAF V600E | 0.0 | 0.0 | Neg |  |  | nt | - |  |  |
| MM483 | 27/02/2017 | BRAF V600E | 0.0 | 0.0 | nt |  |  | nt | - |  |  |
| MM423 | 8/02/2017 | BRAF V600E | 0.0 | 0.0 | nt |  |  | nt | - |  |  |
| MM514 | 12/10/2016 | BRAF V600E | 0.0 | 0.0 | nt |  |  | nt | - |  |  |
| MM469 | 31/08/2016 | BRAF V600E | 0.0 | 0.0 | nt |  |  | nt | - |  |  |
| MM154 | 1/07/2015 | BRAF V600E | 0.0 | 0.0 | nt |  |  | nt | - |  |  |
| MM153 | 15/04/2014 | BRAF V600E | 0.0 | 0.0 | nt |  |  | nt | - |  |  |
| ***Vemurafenib*** |  |  |  |  |  |  |  |  |  |  |  |
| MM035 | 11/04/2013 | BRAF V600E | 174.0 | 6.0 | neg |  |  | nt | - |  |  |
| MM107 | 10/04/2013 | BRAF V600K | 135.0 | 4.9 | neg |  |  | nt | - |  |  |
| MM001 | 27/11/2013 | BRAF V600E | 46.5 | 3.8 | nt |  |  | nt | - |  |  |
| MM056 | 10/11/2014 | BRAF V600E | 14.0 | 0.5 | neg |  |  | nt | - |  |  |
| MM141 | 19/03/2014 | BRAF V600E | 13.5 | 2.0 | neg |  |  | nt | - |  |  |
| MM069 | 10/04/2013 | BRAF V600E | 0.0 | 0.0 | nt |  |  | nt | - |  |  |
| MM143 | 4/09/2013 | BRAF V600E | 0.0 | 0.0 | nt |  |  | nt | - |  |  |
| nt- not tested, * BRAF amplification expressed as a ratio of BRAF copies/VOPP1 copies | | | | | | | | | | |  |

**Supplementary Table 1. Progressive disease samples analysed for the presence of BRAF splicing**
